# Robotic emergency general surgery, future or fallacy?: case-matched comparison of operative and clinical outcomes during the adoption phase in a tertiary centre

**DOI:** 10.1007/s11701-025-02851-y

**Published:** 2025-10-02

**Authors:** Kirsty Cole, Azita Shahdoost-Rad, Youssef Ibrahim, Grace Chaplin, Philip H. Pucher

**Affiliations:** 1 Department of General Surgery, Portsmouth University Hospitals NHS Trust, Portsmouth, UK; 2 Imperial College London, London, UK; 3 Division of Pharmacy and Biosciences, University of Portsmouth, Portsmouth, UK

**Keywords:** Robotics, Emergency general surgery, Minimally invasive, CEPOD

## Abstract

**Aims:**

Robotic surgery continues to expand rapidly in elective settings; however, its role in emergency care is limited to date. This study aims to evaluate the safety and feasibility of the adoption of robotic emergency general surgery (EGS) within a high-volume centre.

**Methods:**

Robotic EGS cases performed between 2020 and 2024 at a large UK university hospital were identified and matched 1:3 to non-robotic cases based on operation type, age, gender, and pathology. Data on demographics, operative details, and operative and clinical outcomes were collected. Groups were compared using appropriate statistical tests.

**Results:**

A total of 369 patients were included, with 95 (25.7%) in the robotic and 274 (74.3%) in the non-robotic (open/laparoscopic) EGS group. There were no differences between groups for demographics, procedures, or pathology. No statistically significant differences were observed in major complications (10.5% vs 9.1%, *p* = 0.688), conversion to open surgery (1.1% vs 3.9%, *p* = 0.174), post-operative length of stay (4 vs 3 days, *p* = 0.814), and 6-month mortality (0.0% vs 2.9%, *p* = 0.092) between robotic and non-robotic groups. Adjusted analyses showed no association between surgical approach and differences in operative time, major complications, or post-operative stay.

**Conclusion:**

The introduction of robotic emergency general surgery is safe and feasible with comparable short-term clinical outcomes to non-robotic approaches. Further research is needed to explore the impact of an established robotic EGS programme on long-term clinical, patient, and surgeon-reported outcomes.

## Introduction

The evidence for, and uptake of, robotic surgery continues to expand in elective surgical settings [1–4]. Despite this, the use of robotic surgical platforms in emergency general surgery (EGS) has been much more limited to date, with an acknowledged evidence gap in this context [5, 6]. This is notable given that EGS accounts for over half of general surgical activity in Western healthcare systems [7, 8]. EGS patients often present with complex, time-sensitive conditions and carry the highest risk of surgical morbidity and mortality [9–11], underscoring the need to explore the role of surgical advances in this setting.

At present, robotic EGS remains nascent, with only 1–2% of EGS cases currently performed robotically [12, 13]. Current literature is largely composed of limited clinical case series [14–17], or administrative database studies that lack granularity [6, 13, 18, 19]. This gap in high-quality data is echoed in a position paper by the World Society of Emergency Surgery (WSES), which states that although current literature suggests robotic surgery is safe and feasible overall, there is currently a substantial lack of evidence to support its use in emergency general surgery procedures [5]. In addition, given the recognised potential for learning curves to impact outcomes [2, 20], early data from centres without established robotic programmes may also negatively influence results, in a manner similar seen in the early days of laparoscopy [21]. However, it has also been shown that with safe training and adoption processes, elective robotic surgery can be introduced with minimal impact on outcomes (18,19).

Robotic EGS may offer certain potential advantages, such as enhanced surgeon ergonomics [22], decreased post-operative length of stay [23], shorter operative times [24] and lower rates of conversion to open surgery [18]. However, these reported effects are highly variable [12, 25, 26], and evidence on laparoscopic and robotic learning curves shows that early adoption can carry risks [21, 27–29]. Moreover, a recent systematic review of over 5 million patients highlighted that the widespread adoption of robotic EGS is impacted by higher costs and a lack of high-quality evidence [30]. Whilst it is hoped that the upfront costs might be amortised through a reduction in complications or length of stay, evidence on this to date is lacking [6].

This study aims to evaluate the safety and feasibility of introducing robotic emergency general surgery (EGS) in a high-volume English tertiary centre.

## Methods

This was a single-centre retrospective analysis of patients undergoing emergency surgery between 2020 and 2024 (inclusive) at a single large tertiary centre. Queen Alexandra Hospital, Portsmouth, is a high-volume tertiary general surgical centre, with a surgical robotics programme currently performing > 1500 robot-assisted cases per year. During the period of the study, the number of robotically trained general surgeons increased from 2 to 13, with a corresponding increase in case volume and use in both elective and emergent settings. Patients who received robot-assisted EGS were selected at the discretion of the surgeon, depending on the equipment availability and presence of appropriately trained staff. All robotic operations were conducted using a daVinci X or Xi platform (Intuitive Surgical, Sunnyvale, CA, USA). Surgeons all underwent a formal training pathway including observation, simulation, dry and wet lab training followed by proctored gradual adoption before being signed off for independent practice. Institutional governance was ensured through a dedicated robotic surgery oversight group, ongoing outcomes monitoring, and routine morbidity and mortality review. All had performed significant volumes of elective colorectal or upper gastrointestinal / esophago-gastric surgery.

Data regarding patient demographics, pathology, operative details, grade of surgeon, and clinical outcomes were retrieved from an institutional electronic database. Each robotic case was matched to three equivalent non-robotic cases. Cases were manually matched to equivalent cases based on potential confounding factors, including operation type, age, pathology severity, and gender, ensuring comparability and minimising bias. Previous studies using similar methodologies typically employed a 1:1 case-matched ratio [31–33]; to improve statistical power and validity, we increased this ratio to 1:3. Both open and laparoscopic procedures were included in the comparator group to ensure that outcomes of robotic EGS were evaluated against the full spectrum of contemporary surgical practice. For cholecystitis and diverticulitis cases, pathology severity (degree of inflammation) was also matched using Nassar and Hinchey scores, respectively [34–36]. Patients under the age of 18 were excluded. Only operations performed during the day were used for this analysis. As defined by the UK intercollegiate surgical curriculum programme (ISCP) [37], cases in which a significant proportion of the operation was performed by a surgical trainee (as indicated on the operation note) were defined as “training cases”. All training cases were supervised by trained consultant robotic surgeons. This analysis was institutionally approved as a service evaluation, comparing anonymised outcome data of existing surgical practices.

Outcome data included operation length, conversion to open surgery, post-operative stay, major post-operative complications (Clavien–Dindo 3 or above), and mortality at 30 days and 6 months. These were compared between the robotic and non-robotic groups using chi-squared and Mann–Whitney tests. Logistic regression analysis was used to assess major complication rates whilst adjusting for patient and disease variables. Indicative linear regression analyses were performed for operation times and length of stay, utilizing dummy variables to account for categorical variables.

Data were analysed using SPSS (version 30, IBM Corp, Armonk, NY, USA). We interpreted the statistical precision of our results using a 95% CI (confidence interval), as well as a *p *value of < 0.05. This study was locally reviewed by the appropriate institutional authority and registered as a retrospective service evaluation of anonymised outcome data.

## Results

During the study period, 95 patients underwent robotic emergency general surgery. These were matched 1:3 to patients receiving non-robotic surgery, with complete data for 274 patients in the non-robotic group included in the final data synthesis. In total, 369 patients were included in the study. The patient demographics and procedure details are described in Table 1. The median age was 61 years, and 52.0% were female. There were no significant differences between groups for demographical, procedure type, or disease severity data.

**Table 1:** Patient demographics, procedure details and pathology severity

		All	Robotic	Non-Robotic	*p* value
*n*		369	95	274	
Age (median, IQR)		61.00 (45.50–74.00)	61.00 (43.00–75.00)	61.50 (45.75–74.00)	0.784
Gender					0.892
	Female	192 (52.0%)	50 (52.6%)	142 (51.8%)	
	Male	177 (48.0%)	45 (47.4%)	132 (48.2%)	
Charlson comorbidity score					0.590
	0	260 (70.5%)	69 (72.6%)	191 (69.7%)	
	1 +	109 (29.5%)	26 (27.4%)	83 (30.3%)	
Procedure group					0.988
	Cholecystectomy	119 (32.2%)	30 (31.6%)	89 (32.5%)	
	Cholecystectomy + CBDE	96 (26.0%)	24 (25.3%)	72 (26.3%)	
	Colectomy	99 (26.8%)	26 (27.4%)	73 (26.6%)	
	Other	55 (14.9%)	15 (15.8%)	40 (14.6%	
Procedure					0.696
	Anterior resection	27 (7.3%)	12 (12.6%)	15 (5.5%)	
	Bypass of malignant obstruction	4 (1.1%)	1 (1.1%)	3 (1.1%)	
	Cholecystectomy	119 (32.2%)	30 (31.6%)	89 (32.5%)	
	Cholecystectomy + CBDE	96 (26.0%)	24 (25.3%)	72 (26.3%)	
	Creation of stoma	4 (1.1%)	1 (1.1%)	3 (1.1%)	
	Gastrectomy	4 (1.1%)	1 (1.1%)	3 (1.1%)	
	Hartmann’s	29 (7.9%)	4 (4.2%)	25 (9.1%)	
	Hiatal or diaphragmatic hernia repair	9 (2.4%)	3 (3.2%)	6 (2.2%)	
	Internal hernia	4 (1.1%)	1 (1.1%)	3 (1.1%)	
	Lap assisted ERCP	1 (0.3%)	1 (1.1%)	0 (0.0%)	
	Segmental colectomy	37 (10.0%)	8 (8.4%)	29 (10.6%)	
	Small bowel resection	8 (2.2%)	2 (2.1%)	6 (2.2%)	
	Subtotal colectomy	6 (1.6%)	2 (2.1%)	4 (1.5%)	
	Ventral hernia	8 (2.2%)	2 (2.1%)	6 (2.2%)	
	Washout/drainage	13 (3.5%)	3 (3.2%)	10 (3.7%)	
Severity of cholecystitis	Nassar 1	55 (27.4%)	12 (29.3%)	43 (26.9%)	0.071
	Nassar 2	88 (43.8%)	12 (29.3%)	76 (47.5%)	
	Nassar 3	38 (18.9%)	13 (31.7%)	25 (15.6%)	
	Nassar 4	20 (10.0%)	4 (9.8%)	16 (10.0%)	
Severity of diverticulitis					0.849
	Hinchey 1	7 (18.4%)	1 (14.3%)	6 (19.4%)	
	Hinchey 2	23 (60.5%)	4 (57.1%)	19 (61.3%)	
	Hinchey 3	7 (19.4%)	2 (28.6%)	5 (16.1%)	
	Hinchey 4	1 (2.6%)	0 (0.0%)	1 (3.2%)	
Training case		83 (25.6%)	20 (29.4%)	63 (24.6%)	0.420

*IQR* interquartile range, *CBDE* common bile duct exploration, *colectomy* any large bowel resection

**Fig. 1:**
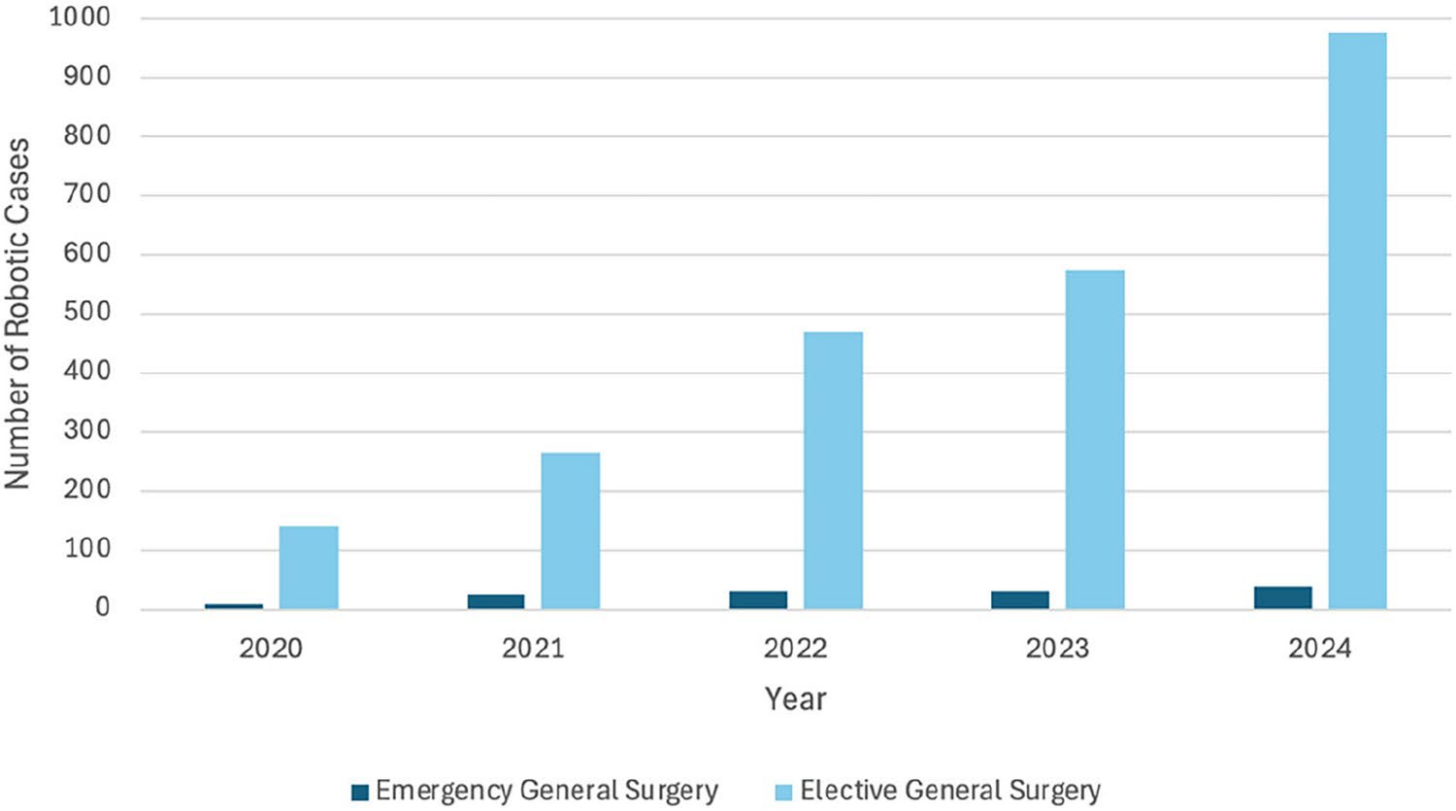
Temporal trend in robotic general surgery case volume from 2020 to 2024

Between 2020 and 2024, the number of consultant general surgeons that were signed off as independent robotic operators increased from 2 to 13 (+ 550%), whilst annual general surgery procedures (elective and emergency) rose from 117 to 1,015 (+ 768%). Across all surgical specialties, robotic surgeons increased from 11 to 34 (+ 209%), and total procedures grew from 361 to 1,555 (+ 331%). The year-on-year increase in robotic general surgery case volume is demonstrated in Fig. 1. Between 2020 and 2024, the number of robotic systems increased from 2 to 4 (2 × da Vinci X and 2 × da Vinci Xi).

Outcome data are presented in Table 2**.** The rate of major complications was similar between the robotic and non-robotic groups (10.5% vs. 9.1%, *p* = 0.688). The details of all complications are reported in Table 3. Only one (1/95, 1.1%) robotic case was converted to open surgery compared to 10/260 (3.8%) in the non-robotic group, though this was not statistically significant (*p* = 0.174). The median post-operative length of stay was 4 days in the robotic group and 3 days in the non-robotic group (*p* = 0.814). No deaths occurred within six months in the robotic group, whereas eight deaths (2.9%) were reported in the non-robotic group (*p* = 0.092). 2/8 deaths were due to post-operative cardiac arrests; 6/8 not felt to be related to the emergency operation.

**Table 2:** Outcome data

		All	Robotic	Non-Robotic	*p* value
Operative times by operation (minutes, median (IQR))	All	96 (65–150)	120 (75–217)	90 (64.25–135)	< 0.001
	Cholecystectomy	71 (60–90)	60 (47–91)	74 (60–90)	0.449
	Cholecystectomy + CBDE	96 (80–120)	120 (90–128)	90 (75–120)	0.037
	Nassar 1–2	80 (60–105)	90 (60–114)	80 (60–105)	0.921
	Nassar 3–4	88 (74–120)	105 (75–130)	80 (68–100)	0.071
	Colectomy (all)	195 (140–255)	266 (217–305)	170 (120–208)	< 0.001
	Hinchey 1–2	150 (105–240)	240 (224–331)	145 (85–184)	0.006
	Hinchey 3–4	175 (139–251)	271 (240–301)	160 (131–199)	0.143
	Other	86 (60–127)	125 (95–240)	68.5 (60–120)	0.003
Initial approach	Robotic Laparoscopic Open	95 264 10	95 (100%) – –	- 260 (94.9%) 14 (5.1%)	–
Conversion to open		11 (3.1%)	1/95 (1.1%)	10/260 (3.8%)	0.174
Complication	Any	57 (15.4%)	18 (18.9%)	39 (14.2%)	0.273
	Major (CD3 +)	35 (9.5%)	10 (10.5%)	25 (9.1%)	0.688
Post-operative LOS		3.00 (1.00–7.00)	4.00 (2.00–6.00)	3.00 (1.00–7.00)	0.814
30-day mortality		3 (0.8%)	0 (0.0%)	3 (1.1%)	0.306
6-month mortality		8 (2.2%)	0 (0.0%)	8 (2.9%)	0.092

IQR  interquartile range, *CBDE* common bile duct exploration, *LOS * length of stay, *colectomy*  any large bowel resection

**Table 3:** Post-operative complications

CD Grade	All	Robot	Non-robotic
**CD 1**	**5**	**0**	**5**
AKI	2	0	2
Chronic pain	1	0	1
VTE	1	0	1
Ileus	1	0	1
**CD 2**	**17**	**8**	**9**
Intra-abdominal collection/haematoma	5	2	3
Wound infection	4	2	2
VTE	2	0	2
HAP	1	0	1
Bladder injury	1	1	0
Ileus	2	1	1
Adverse drug reaction	1	0	1
Other infection	3	3	0
**CD 3**	**31**	**10**	**21**
Bile leak	3	1	2
Retained Stone	13	5	8
Bleed	1	1	0
Intra-abdominal collection/haematoma	9	2	7
Duodenal stump leak	1	0	1
Wound complication	2	0	2
Lung abscess	1	1	0
Hernia recurrence	1	0	1
**CD 4**	**2**	**0**	**2**
Cardiac Arrest	1	0	1
Retained stone—biliary sepsis	1	0	1
**CD 5**	**2**	**0**	**2**
Cardiac Arrest	2	0	2
**Any complication**	**57**	**18**	**39**

*CD* clavien–dindo, *AKI *acute kidney injury, *VTE*  venous thromboembolism, *HAP*  hospital acquired pneumonia

There was no significant difference in operative time for cholecystectomy (*p* = 0.449). Unadjusted operative times were longer for robotic cholecystectomy, which included common bile duct exploration (CBDE) (*p* = 0.037) and colectomy (*p* < 0.001); however, in a linear regression analysis adjusting for patient demographics, procedure type, and surgeon grade, surgical approach was not significantly associated with operative time (OR 0.06 (95% CI − 8.10–27.23), *p* = 0.274) (Appendix Table 5).

Similarly, in adjusted analyses, the robotic approach did not significantly affect the rate of major complications (OR 1.47 (95% CI 0.63–3.46), *p* = 0.337) (Table 4) or the length of post-operative stay (OR − 0.02 (95% CI − 2.10–1.59), *p* = 0.774) (Appendix Table 6).

**Table 4:** Logistic regression analysis for major post-operative complications

Covariate		OR (95% CI)	p value
Robotic approach		1.47 (0.63–3.46)	0.337
Age		0.99 (0.97–1.02)	0.621
Male gender		1.59 (0.69–3.53)	0.288
Charlson morbidity score 1 or above		1.38 (0.57–3.35)	0.482
Procedure group	Cholecystectomy	Ref	
	Cholecystectomy + CBDE	0.75 (0.27–2.07)	0.576
	Colectomy	0.11 (0.01–0.89)	0.038
	Other	2.06 (0.80–5.26)	0.137
Training case		0.64 (0.24–1.68)	0.360

*CBDE*  common bile duct exploration, *colectomy*  any large bowel resection

## Discussion

This study is one of the largest to date to compare robotic and conventional EGS in a matched patient group, including patient demographics, pathology/indication for surgery, and disease severity. This multivariable analysis found no significant differences in efficiency (operative times) or clinical outcomes during the adoption/expansion phase for robotic surgery in a high-volume centre. Conversion rates were noticeably lower although not statistically significant (1.1% vs 3.8%, *p* = 0.174). Overall, these data suggest robotic EGS can be feasibly and safely delivered, even within the increasingly resource-constrained environment of the UK healthcare system.

This study adds significantly to the existing literature on the utility of robotic EGS, which remains of variable size and quality. Whereas some studies have reported negative clinical outcomes in some robotic EGS contexts such as cholecystectomy [38, 39], larger systematic reviews [6] or national database studies [13] have not corroborated these findings, instead suggesting that outcomes are equivalent or even superior to traditional approaches.

Conversion to open surgery was shown to be at least equivalent in our cohort, consistent with the findings reported by Klein et al. [19]. Notably, several studies have reported lower conversion rates with robotic approaches. For example, Lunardi et al. found reduced conversion in robotic versus laparoscopic procedures: cholecystectomy (1.7% vs. 3.0%), colectomy (11.2% vs. 25.5%), inguinal hernia repair (2.4% vs. 10.7%), and ventral hernia repair (3.5% vs. 10.9%) [18]. Similarly, Rifai et al. demonstrated lower conversion rates for robotic appendectomy and cholecystectomy [23].

Regarding operative time, our adjusted data demonstrated no significant difference between robotic and non-robotic procedures. This is consistent with findings by Rifai et al. [23], whilst Klein et al. even observed a reduction in operative time in the robotic cohort [19]. While not statistically significant, we note that median operative time for higher volume, less complex procedures such as cholecystectomy was shorter in the robotic group but higher for other procedures, suggesting the presence of a learning curve bias which might be expected to change with increased volume and experience. Data volume and case mix heterogeneity precluded formal learning curve analysis at the surgeon level.

Minimally invasive EGS is already known to reduce post-operative mortality and hospital stay compared to open surgery [40, 41]. It is reasonable to expect that a surgical robotic platform offering enhanced dexterity, vision, and technology can enhance these benefits through improved rates of successful minimally invasive surgery (reduced conversions) and greater surgical precision [42, 43]. However, confirming these advantages will likely require large-scale datasets with more granular detail, with greater consistency of case mix and surgical expertise.

In the UK, as in many other Western countries, most general surgeons currently remain in the early adoption or learning curve phase of robotic surgery [44], which could attenuate the observable benefits of the approach—similar to the historical transition to laparoscopy [21]. As visualised in Fig. 1, the centre underwent significant expansion of robotic general surgery from 2020 to 2024, together with an increase in available robotic systems (two to four). Nevertheless, the annual volume of robotic EGS procedures seen in this series remains relatively low, highlighting the practical challenges of implementing such a programme. Successful adoption requires access to appropriately trained staff and institutional approval, particularly given the increased cost of disposables [45]. Additionally, because dedicated robotic platforms for EGS do not currently exist in the UK, emergency cases could only be performed when the systems were not in use for elective surgery.

Importantly, despite these logistical and systemic constraints, our data demonstrate that emergent robotic procedures can be performed safely, supporting the feasibility of expanding robotic access. These findings suggest that with structured training, exposure, and institutional support, robotic EGS can be introduced without significant impact on hospital efficiency or patient outcomes, and that further improvements may become apparent as the learning curve is surpassed.

Policymakers are now increasingly recognising the need to introduce the latest surgical technologies, albeit in a safe and controlled manner [46] that does not negatively impact existing clinical care. The data from this study suggest that with appropriate training, exposure, and support, robotic EGS can be introduced without significant impact on hospital efficiency or patient outcomes. Potential concerns regarding complications associated with a new surgical approach, or significant impact on training opportunities, were not justified here. With the increasing adoption of robotic EGS, and as the learning curve is achieved and surpassed, it may be that greater differences in efficiency and outcomes become more notable.

Despite its strengths, this study is subject to certain limitations. Whilst our study evaluated major complications and mortality at 30 days and 6 months, long-term outcomes were not captured. Further investigation into these outcomes could provide valuable insights. Moreover, although comorbidity scores were accounted for, additional data on patient selection factors, such as BMI and preoperative physiological status, could further reduce potential for selection bias and evaluate alignment of case selection with WSES guidelines [5].

Beyond these clinical considerations, there are additional factors that warrant attention. Patient preferences represent an important but understudied aspect of robotic EGS, and understanding how patients perceive and value robotic interventions could enhance shared decision-making. Additionally, the influence of robotic surgery on cognitive workload remains unclear. Further research in this area could yield strategies to enhance surgical performance and team dynamics.

In summary, this study supports the introduction of robotic EGS, with outcomes comparable to traditional approaches. Whilst limitations, including dataset heterogeneity and a lack of long-term data, exist, these findings lay a strong foundation for future research and suggest that the adoption of routine robotic EGS is feasible and safe. Further studies should explore long-term outcomes, patient-reported experiences, and the cognitive demands of robotic surgery to better understand its role in emergency general surgery.

## Data Availability

No datasets were generated or analysed during the current study.

## Appendix

See Tables 5 and 6

**Table 5:** Linear regression for operation length (minutes)

Covariate		OR (95.% CI)	*p* value
Robotic approach		0.06 (− 8.10–27.23)	0.287
Age		0.09 (− 0.11–0.79)	0.142
Gender		− 0.24 (− 46.96–17.61)	< 0.001
Charlson morbidity score		− 0.04 (− 13.26–7.07)	0.550
Training case		− 0.07 (− 27.96–5.11)	0.175

Dummy variables used for categorical variables

**Table 6:** Linear regression for post-operative length of stay (days)

Covariate		OR (95.% CI)	*p* value
Robotic approach		− 0.02 (− 2.10–1.59)	0.789
Age		0.21 (0.04–0.13)	< 0.001
Gender		− 0.07 (− 2.45–0.59)	0.228
Charlson morbidity score		0.02 (− 0.85–1.27)	0.693
Training case		− 0.08 (− 2.98–0.47)	0.155

Dummy variables used for categorical variables
